# Non-linear Dynamic Shifts in Distress After Wildfires: Further Tests of the Self-Regulation Shift Theory

**DOI:** 10.3389/fpsyg.2020.551962

**Published:** 2020-10-06

**Authors:** Charles C. Benight, Kotaro Shoji, Aaron Harwell, Erika Felix

**Affiliations:** ^1^National Institute for Human Resilience, University of Colorado, Colorado Springs, Colorado Springs, CO, United States; ^2^Department of Psychology, University of Colorado, Colorado Springs, Colorado Springs, CO, United States; ^3^Department of Counseling, Clinical, & School Psychology, University of California, Santa Barbara, Santa Barbara, CA, United States

**Keywords:** wildfire, trauma, social cognitive theory, self-regulation shift theory, cusp catastrophe, coping self-efficacy, perceived loss, peritraumatic dissociation

## Abstract

Worldwide exposure to explosive wildfires has become increasingly common. The psychological impact of these fires is substantial, demanding a deeper understanding of post-wildfire adaptation. This paper consists of two studies aiming to test self-regulation shift theory and its predicted non-linear shifts in distress using cusp catastrophe analyses. Study 1 tested a cusp catastrophe model on distress after the Waldo Canyon wildfire, Colorado (June, 2012). Results of study 1 showed that coping self-efficacy early after the wildfire was a significant bifurcation factor affecting when a shift in distress levels occurred from a lower state to an upper state. Perceived loss was a significant asymmetry controlling factor affecting the relative strength of each state. These findings indicate that a non-linear shift is more likely to occur at lower levels of coping self-efficacy and higher perceived loss. Study 2 tested the same model among survivors of several wildfires in California during 2017 and 2018. Results of study 2 confirmed the importance of coping self-efficacy again as a significant bifurcation factor. In this case, peritraumatic dissociation was found to be a significant asymmetry controlling factor instead of loss. These results indicate that an upward shift in distress occurs when coping self-efficacy is lower and peritraumatic dissociation is higher. Collectively, the combined findings suggest that coping self-efficacy is a pivotal variable consistent with self-regulation shift theory predictions. Intervention implications are discussed.

## Introduction

Mega wildfires are a worldwide problem with significant social, economic, and ecological consequences (Gill et al., 2013). A recent congressional research report showed that every year since 2000, the average acreage burned in the United States is nearly double the loss during the 1990s (Congressional Research Service, 2019). The number of individuals suffering from wildfires has been increasing as well (National Interagency Fire Center, 2018). Among survivors of wildfires, 24% met probable diagnosis of posttraumatic stress disorder (PTSD) and 33% met probable diagnosis of major depression 3 months after the October 2003 California firestorm (Marshall et al., 2007). Following the 2007 Peloponnese wildfire in Greece, 46.7% of survivors were diagnosed with PTSD after 1 month after the 2007 and 29.4% of adolescents met the criteria for probable PTSD 6 months after the wildfire (Papadatou et al., 2012; Psarros et al., 2017). Despite the noted aversive consequences of disasters (e.g., PTSD, depression, and anxiety), most individuals exposed to natural disasters do not experience clinically significant psychological distress or impairment (Norris et al., 2002; Galea et al., 2005; Neria et al., 2008). Over the past several decades, the work of disaster researchers converged to highlight many factors that influence the likelihood or course of post-disaster distress, including low self-efficacy, female gender, greater concurrent stressors, higher levels of disaster exposure, low social support, and prior psychiatric history (Green, 1998; Benight et al., 1999; Norris et al., 2002; Benight and Bandura, 2004; Galea et al., 2005; Norris, 2005; North et al., 2012; North and Pfefferbaum, 2013). Despite this information, we still do not have detailed theoretical or empirical information that helps to explain the dynamic adaptation process associated with disaster recovery (Benight and McFarlane, 2007). This paper attempts to help fill this void by reporting on two studies testing self-regulation shift theory (SRST) that predicts key mechanisms for non-linear shifts (i.e., discontinuous symptom acceleration) in distress during the disaster recovery process.

Self-regulation shift theory is based on social cognitive theory. Social cognitive theory provides a framework that explains the cognitive, affective, and motivational processes involved with human adaptation following disasters and trauma (Benight and Bandura, 2004). Traumatic stress adaptation can be understood by describing the bidirectional, dynamic interactions among individual’s psychological variables (e.g., cognitive, affective) and behavioral and social contextual variables (Benight and Bandura, 2004). Self-efficacy is a critical self-appraisal person factor that guides coping processes through self-evaluation. Coping self-efficacy (CSE) is a self-appraisal of one’s capability for coping with demands and challenges in a stressful situation that directly ties to the management of traumatic stress demands (Benight and Bandura, 2004). It is predictive of coping following a variety of potentially traumatic events including natural disasters such as wildfires (Benight and Harper, 2002), hurricanes (Benight et al., 1999; Hirschel and Schulenberg, 2009; Wadsworth et al., 2009), earthquakes (Guerra et al., 2014), and floods (Pritchard and Gow, 2012). For review, see Benight and Bandura (2004) and Luszczynska et al. (2009).

Furthermore, longitudinal research on disaster survivors (Benight and Harper, 2002; Bosmans et al., 2013) showed that CSE plays a prime mediating role in disaster recovery (Benight et al., 1999). Collectively, this research supports the important influence that self-evaluation has on coping success and failure within a disaster recovery context. This ability is central in providing feedback necessary for recalibration of coping efforts as the recovery unfolds. Whereas these results demonstrate the importance of self-appraisals in trauma and disaster adaptation, they do not provide information on the dynamic process of disaster stress adaptation. Self-regulation shift theory offers a theoretical extension of social cognitive theory to help explain this dynamic process.

Self-regulation shift theory (Benight et al., 2017) is a theory of motivation where self-determination is a central component. Deci and Ryan (2000) highlighted the basic human need for self-determination defined as the intrinsic motivation to have influence over personally relevant environmental conditions that affect one’s well-being. Within the context of disaster recovery, the internal drive to regain a sense of normalcy is front and center to survivors and provides a motivational surge to put life back together.

Self-regulation shift theory argues that the self-regulation feedback process outlined in social cognitive theory drives critical self-evaluative judgments during recovery that enable humans to adjust to an ever changing recovery landscape (e.g., dealing with insurance companies, finding temporary housing, etc.). However, based on SRST, for some survivors, this feedback process can hit a self-determination threshold where the perceived ability to manage the post-disaster recovery and regain a sense of control is shattered. Self-regulation shift theory refers to this threshold as a coping “tipping point” called the *self-determination violation effect*. Once reached, a fundamental shift occurs, resulting in a non-linear or rapid acceleration of negative cognitive, motivational, social, affective, and behavioral outcomes. Self-regulation shift theory is unique relative to other traumatic stress theories in that it targets the identification of key catalyst variables related to this non-linear systemic change or shift across time. A non-linear dynamic systems approach provides a new perspective into how survivors’ unfolding coping processes operate under certain hypothesized conditions.

The four primary tenets of SRST are as follows: (1) Human beings are self-aware dynamic living systems that have the ability to utilize internal and external feedback to self-regulate toward desired goals (Bandura, 1997; Ford, 1987). (2) Under certain conditions, living systems can be pushed into non-linear dynamic shifts from one organized state to another based on environmental and internal pressures. (3) Coping response output after trauma or disaster comprises a biopsychosocial action relative to the perceived level of disequilibrium (or distance from a state of normalcy) combined with one’s belief in being able to manage effectively this discrepancy. (4) A subset of survivors reach a critical threshold when they believe it is just not possible to regain a sense of control over their recovery, a state referred to as the self-determination violation effect. When individuals reach this critical threshold, the system reorganizes into a new state of the impaired self (i.e., a new negative systemic equilibrium).

**FIGURE 1:**
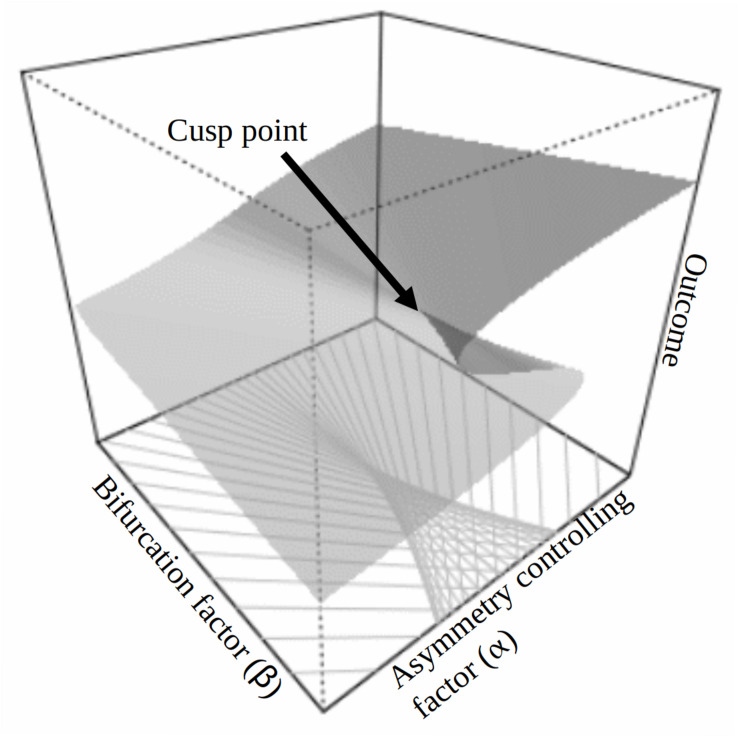
Cusp catastrophe model.

Evidence exists supporting SRST and the non-linear negative shifts that are hypothesized during trauma adaptation. Benight et al. (2017) found support for negative non-linear shifts in functioning 3 months after a motor vehicle accident (MVA) in two separate samples. Cusp catastrophe models were used to test for non-linear dynamic shifts in posttraumatic distress. In cusp catastrophe modeling, bifurcation factors and asymmetry controlling variables are used to predict the non-linear shift (see Figure 1). The bifurcation factor (β) determines when a cusp happens, and an asymmetry controlling factor (α) affects the relative strength of each state’s attractiveness. CSE served as a critical catalyst variable (i.e., bifurcation factor) for the negative non-linear shift in posttraumatic symptoms in both MVA samples. Importantly, those who reported *less*, rather than more, peritraumatic dissociation during the accident (sample 1) or showed less injury (sample 2) were more likely to be in a higher state of distress at 3 months, relative to CSE perceptions. Specifically acute (7 days) and subsequent (1 month) lower CSE beliefs served to bifurcate the outcome at 3 months into a non-linear higher distress plane or lower distress plane. Asymmetry controlling factors or “context” parameters are expected to impact the outcome variable relative to the bifurcation variable. Theoretically, we hypothesized that CSE perceptions would generalize across trauma types (e.g., MVA to natural disaster), but specific contextual factors may be important to measure for asymmetry controlling variables (e.g., damage and loss from the disaster). Collectively, the MVA results suggested that SRST offers a new lens for understanding critical mechanisms associated with unique trajectories of coping with traumatic stress. The present paper provides a further test of SRST beyond the motor vehicle trauma context with two separate wildfire disaster samples.

### Present Study

A major wildfire disaster pushes individuals out of a steady-state equilibrium, resulting in significant energy (i.e., coping output) being allocated to rebuilding and establishing a sense of normalcy again. These massive fires radically change survivors’ lives often within a matter of hours. Homes are destroyed, the landscape obliterated, and lives lost. We focused on two separate wildfire disasters for the present paper.

The Waldo Canyon Wildfire occurred in June 2012. It raged across the foothills directly west of Colorado Springs, CO, burning over 18,247 acres, destroying 346 homes, and killing 2 people. The fire resulted in the evacuation of 32,000 residents (Denver Post, 2012; Linton, 2012; St. Louis-Sanchez, 2012). Participants of this study were residents living in the affected area at the time of the wildfire. Because Benight et al. (2017) identified injury severity as an asymmetry controlling factor, we used perceived loss that can be considered a measure of the wildfire severity.

The second wildfire disaster comprised five different fires across California during the unprecedented 2017–2018 year (Thomas Fire, Holiday Fire, Carr Fire, Camp Fire, and Woolsey Fire). The Thomas Fire started in December 2017 and burned 281,893 acres (InciWeb-Incident Information System, 2018). It destroyed 1063 structures and damaged 280 structures. The Holiday Fire started in July 2018, burned 113 acres, and destroyed 28 structures [California Department of Forestry and Fire Protection, 2019c]. The Carr Fire also started in July 2018, burned 229,6521 acres, destroyed 1614 structures, damaged another 61 structures, and involved 3 fatalities (California Department of Forestry and Fire Protection, 2019b). The Camp Fire, which started on November 08, 2018, was the most destructive wildfire in California history at the time of data collection. It burned 153,336 acres and virtually destroyed the entire community of Paradise, CA, United States. The Camp Fire resulted in 3 injuries and killed 85 civilians and cost an astounding 16.5 billion dollars in damage (California Department of Forestry and Fire Protection, 2019a). Finally, the Woolsey Fire started in November 2018, burned 96,949 acres, destroyed 1643 structures, damaged 341 structures, and resulted in 3 fatalities (California Department of Forestry and Fire Protection, 2019d). This second sample included individuals who were exposed to one of these five fires. Benight et al. (2017) found peritraumatic dissociation as a significant asymmetry controlling factor; thus, we used peritraumatic dissociation as a potential asymmetry controlling factor in our models as well.

## Study 1: Waldo Canyon Fire

Study 1 was part of a larger randomized trial on a web intervention for disaster recovery following the Waldo Canyon Fire. Participants of this study were residents living in the affected area at the time of the wildfire. Based on the MVA study, we hypothesized that CSE perceptions would serve as a key catalyst or bifurcation factor in predicting a non-linear shift in functioning supporting SRST. Previous studies have identified CSE as a bifurcation factor for posttraumatic distress among people suffering from an MVA (Benight et al., 2017), condom-use intention (Yu et al., 2018), and post-treatment alcohol use (Witkiewitz et al., 2007).

The asymmetry controlling variables we focused on were extent of damage/loss and time since the disaster. Perceived loss of resources due to a traumatic event is a reliable predictor for posttraumatic distress (Hobfoll, 1989, 1991; Finkelstein, 2016). For example, higher perceived loss is related to higher psychological distress among people who experienced Hurricane Hugo (Freedy et al., 1994). Psychosocial loss mediated the relationship between flood exposure and both psychological distress and physical symptoms 6 months after a flood in the United States Midwest (Smith and Freedy, 2000). Based on these findings from the previous studies, we hypothesized that higher perceived loss would be related to greater attractiveness in the higher distress state. We also hypothesized that shorter time since the disaster would be related to greater attractiveness in the higher distress state because distress following a traumatic event tends to be higher shortly after the event than long after the event is over.

### Method

#### Participants

This study was part of a larger study investigating the effectiveness of a web-based intervention, Mydisasterrecovery.com for survivors of a natural disaster. Participants were living in or around Colorado Springs, CO, United States at the time of the wildfire. A total of 189 participants [*M*_*age*_ = 46.68 (*SD* = 14.66), 68.8% female] completed the time 1 assessment, 155 completed the time 2 assessment, and 123 completed the last assessment.

Most participants were Caucasian (93.7%). Other ethnicity of participants included African American (1.6%), Hispanic (1.6%), mixed (1.6%), and other (1.6%). Over a half of participants were married (66.7%), 14.8% have never been married, 9.5% were divorced, 4.2% had a domestic partner, and 4.2% were widowed. Participants’ education levels were relatively high (36.5% graduated from a 4-year college, 36.5% graduated from a graduate school, 19.6% had some college, 3.2% had some high school, and 3.2% graduated from a high school). The median annual income was $100,000 (*SD* = 66,766). Most participants (95.2%) evacuated from home. Results of a Mann–Whitney *U* test showed that there was no statistical difference between evacuees and non-evacuees in T1 distress, *U* = 739, *p* = 0.678; T1 CSE, *U* = 788, *p* = 0.915; and T1 loss, *U* = 939, *p* = 0.404. Thus, we combined the evacuees and non-evacuees in the further analyses.

#### Measures

The primary measures for this specific analysis included distress, trauma CSE, and loss at all three time points, and time since the disaster.

##### Distress

A modified version of the PTSD Checklist-Civilian Version for DSM-IV (PCL; Weathers et al., 2013) assessed posttraumatic distress (e.g., intrusive thoughts, avoidance, and hyper-arousal) due to the wildfire. This 17-item measure assesses post-traumatic stress reactions for the past month on a five-point scale ranging from 1 (*not at all*) to 5 (*extremely*). Due to an administrative error, only 10 items were included. The seven items assessing hyper-arousal were not included. Given that our goal was to assess posttraumatic distress reactions, not to provide a diagnosis, we think that the 10 items provide a reasonable measure of posttraumatic distress. Sample items included “Repeated, disturbing dreams of the wildfire” and “Feeling very upset when something reminded you of the fires.” Cronbach’s alpha coefficients were excellent at 0.87 Time 1 (T1), 0.89 Time 2 (T2), and 0.88 Time 3 (T3). Because we used 10 items, mean scores were calculated instead of conventional total scores.

##### Trauma CSE

Trauma Coping Self-Efficacy Scale (CSE-T; Benight et al., 2015) was used to measure perceived capability to deal with uncertainty and challenges associated with the wildfire. The CSE-T consists of nine items with a seven-point scale ranging from 1 (*not at all capable*) to 7 (*very capable*). Sample items included “Not ‘lose it’ emotionally” and “Get my life back to normal.” Cronbach’s alpha coefficients were 0.90 at T1, 0.91 at T2, and 0.91 at T3.

##### Loss

Perceived loss due to the wildfire was measured using the Conservation of Resources-Evaluation (COR-E; Hobfoll, 1989). The original COR-E was a 74-item measure that assessed material, social, financial, and psychological loss due to the wildfire using a five-point scale, ranging from 0 (*not at all/not applicable*) to 4 (*to a great degree*). We chose 40 items that were potentially related to the wildfire more than other items to reduce the burden of participants. Sample items included “Adequate income” and “Health of family member or close friend.” Cronbach’s alpha coefficient was 0.94 at T1, 0.95 at T2, and 0.95 at T3.

##### Demographics

Participants’ demographic information including age, gender, ethnicity, education, annual income, evacuation status, and time since the wildfire was collected.

#### Procedures

The study was approved by the Institutional Review Board at the authors’ institution. Participants were recruited 63.2 days (*SD* = 9.9) after the wildfire on average through print media, TV, and community response email list-serves. Participants were invited to complete the pre-test survey (T1) and were randomly assigned into an experimental group (a web intervention) or a waitlist control group. Participants completed the post-test online survey approximately 30 days after the pre-test (T2) and the follow-up online survey approximately 60 days after the pre-test (T3). A 3 × 2 mixed ANOVA with assessment periods (T1, T2, and T3) as a within-subjects variable and the group assignment (experimental vs. control) as a between-subjects variable was run to test whether distress levels differed between these two groups across assessment periods. Results showed that there was a significant effect for assessment periods, *F*(2,186) = 15.93, *p* < 0.001, $\eta_{\text{p}}^{2}$ = 0.15; however, the interaction between time and group assignment was not significant, *F*(2,186) = 0.93, *p* = 0.396, $\eta_{\text{p}}^{2}$ = 0.01. Thus, we combined these two groups in further analyses.

#### Data Analysis

We used a series of three polynomial regression analyses for cusp catastrophe to test whether a cusp occurred in distress using a statistical software R (Guastello, 1982, 1987). In the first analysis, residualized scores between T1 distress and T3 distress were calculated and used as a dependent variable. A *z* term was calculated by T1 distress scores minus the minimum score of T1 distress divided by *SD* of T1 distress. *z*^2^ and *z*^3^ were also calculated from the *z* term. These *z* terms represent the system’s state variables or the status of a behavior (Grasman et al., 2009; Xu et al., 2017). A beta value (bifurcation factor) was computed by the multiplication of *z* and T1 CSE. We included T1 loss, gender, and time since the wildfire as alpha values (asymmetry controlling factors) in the model.

Next, we tested whether a cusp in distress occurred between T1 and T2. Residualized scores between T1 distress and T2 distress were calculated as a dependent variable. The same *z*^3^, *z*^2^, beta, and alpha variables as in the first analysis were included in the model.

The third polynomial regression analysis tested whether a cusp in distress occurred between T2 and T3. Residualized scores between T2 distress and T3 distress were computed and used as a dependent variable. A *z* term was calculated by T2 distress minus the minimum T2 distress score divided by *SD* of T2 distress. *z*^3^ and *z*^2^ were calculated from the *z* term. Beta was computed by the multiplication of *z* and T2 CSE. The model included *z*^3^, *z*^2^, beta, and T2 loss, gender, and time since the wildfire (alpha variables) as independent variables.

Finally, we tested whether the cusp models were superior to a linear regression model. For each polynomial regression analysis, a linear regression analysis was run with the same dependent variable as in the polynomial regression analysis. Independent variables included T1 CSE, T1 loss, gender, and time since the wildfire as predictors for the first and second analyses. Independent variables for the third analysis included T2 CSE, T2 loss, gender, and time since the wildfire as predictors. To compare between the cusp models and the linear models, we followed and applied a procedure for multimodel inference proposed by Burnham et al. (2011) to compare multiple models using AIC. We used BIC to help the model selection as well because BIC is a commonly used fit index. A model with a smaller AIC value was considered superior to the other model. As a general rule, a difference in AIC greater than 2 is considered a meaningful difference between two models. A difference in AIC greater than 4 is a moderately meaningful difference, and a difference in AIC greater than 10 means a considerable difference between two models (Burnham and Anderson, 2004; Burnham et al., 2011). Similarly, a model with a smaller BIC is interpreted as a better model (Kass and Raftery, 1995). A BIC difference between 0 and 2 should be interpreted as not worth more than a bare mention. A BIC difference between 2 and 6 means a positive difference. A BIC difference between 6 and 10 indicates a strong difference, and a BIC difference greater than 10 means a very strong difference.

#### Missing Data

In total 0.58, 0.30, and 0.44% of the data were missing data at T1, T2, and T3, respectively. These missing data were imputed using an inverse non-linear principal component analysis method with an R package “pcaMethods” (Stacklies et al., 2007). We used a non-linear imputation method to maintain non-linearity of the data.

### Results

#### Attrition Analysis

Attrition analyses showed that there were no differences in T1 CSE, loss, age, and income between those who completed T2 assessment and those who dropped out. However, dropouts (*M* = 1.22) had higher T1 distress scores than completers (*M* = 0.85), *t*(44.51) = 2.40, *p* = 0.021. There were no differences in T2 CSE, T2 PTS, T2 loss, age, and income between completers of T3 assessment and dropouts. Thus, these analyses should be considered relative to the sample of less distressed individuals.

#### Bivariate Relationships

Pearson’s correlations among the study variables showed that CSE and distress had a negative and large relationship across all three time points (Table 1). CSE had medium to large negative relationships with loss across all three time points. There were medium to large positive relationships between distress and loss across all time points. Finally, time since the wildfire had a positive and small effect with T1 CSE.

**TABLE 1:** Pearson’s correlations, mean, and standard deviations of the study variables in Study 1.

	**1**	**2**	**3**	**4**	**5**	**6**	**7**	**8**	**9**	**10**
(1) T1 distress										
(2) T2 distress	0.83**									
(3) T3 distress	0.81**	0.81**								
(4) T1 CSE	−0.73**	−0.66**	−0.70**							
(5) T2 CSE	−0.76**	−0.78**	−0.75**	0.78**						
(6) T3 CSE	−0.69**	−0.71**	−0.80**	0.75**	0.81**					
(7) T1 loss	0.49**	0.45**	0.50**	−0.37**	−0.44**	−0.43**				
(8) T2 loss	0.51**	0.57**	0.60**	−0.44**	−0.58**	−0.54**	0.78**			
(9) T3 loss	0.48**	0.58**	0.65**	−0.39**	−0.53**	−0.56**	0.78**	0.89**		
(10) Time since	–0.12	–0.09	–0.04	0.18*	0.06	–0.04	–0.14	–0.03	0.05	
Mean	0.91	0.73	0.69	5.37	5.58	5.68	0.89	0.76	0.72	63.16
*SD*	0.75	0.71	0.70	1.16	1.16	1.16	0.64	0.63	0.64	9.88

T1, time 1; T2, time 2; T3, time 3; CSE, coping self-efficacy; SD, standard deviation. **p < 0.001, *p < 0.05.

#### Polynomial Catastrophe Cusp

##### Time 1 and time 2 cusp

We ran a polynomial regression cusp model for residualized scores between T1 (45 days since the disaster) distress and T2 (2.5 months since the disaster) distress (*n* = 155). T1 CSE was used as a bifurcation factor, and T1 loss, gender, and time since the wildfire were used as asymmetry controlling factors. *z*^2^ and *z*^3^ were entered in the model as well. Results showed that *z*^2^ and *z*^3^ were not significant; thus, *z*^3^ was removed from the model. Results of the modified model showed that both asymmetry controlling factors were not significant. Thus, although there was evidence of the cusp in distress with significant *z*^2^ between T1 and T2, the model was not supported (Table 2). AIC and BIC for the final model were 160.51 and 181.82, respectively, and adjusted *R*^2^ was 0.02.

**TABLE 2:** Standardized coefficients, standard error, and *p*-values for the cusp model for Study 1.

**DV**	**IV**	**β**	**Std. error**	***p***
****Δ** distress T1–T2**				
	*z*^2^	0.12	0.05	0.027
	T1 CSE × *z*	–0.14	0.05	0.011
	T1 loss	0.05	0.04	0.212
	Time since	–0.00	0.04	0.964
	Female gender	–0.02	0.07	0.818
****Δ** distress T1–T3**				
	*z*^2^	0.12	0.05	0.026
	T1 CSE × *z*	–0.22	0.06	< 0.001
	T1 loss	0.13	0.04	0.004
****Δ** distress T2–T3**				
	*z*^2^	0.06	0.05	0.225
	T2 CSE × *z*	–0.17	0.05	0.002
	T2 loss	0.13	0.04	0.002
	Time since	0.01	0.04	0.77
	Female gender	0.08	0.08	0.280

*DV, dependent variable; IV, independent variable; β, standardized coefficient; T1, time 1; T2, time 2; Std. error, standard error; CSE, coping self-efficacy.*

##### Time 2 to time 3 cusp

Next, we tested a polynomial regression cusp model for residualized scores between T2 (2.5 months) distress and T3 (3.5 months) distress with T2 CSE (T2 CSE × *z*) as a bifurcation factor and T2 loss, gender, and time since the wildfire as asymmetry controlling factors (Table 2; *n* = 123). Results showed that *z*^2^ and *z*^3^ were not significant; thus, *z*^3^ was removed from the model. Even after removing *z*^3^, *z*^2^ was not significant, indicating that the cusp did not occur in distress between T2 and T3. AIC and BIC for the final model were 124.81 and 144.50, respectively, and adjusted *R*^2^ was 0.09.

##### Time 1 to time 3 cusp

Finally, we ran a polynomial regression cusp model with residualized scores between T1 (45 days) distress and T3 (3.5 months) distress (*n* = 123). We used T1 CSE (T1 CSE × *z*) as a bifurcation factor and T1 perceived loss, gender, and time since the wildfire as asymmetry controlling factors. The *z*^2^ and *z*^3^ terms were also included in the model. Results showed that *z*^2^ and *z*^3^ terms were not significant; thus, we dropped *z*^3^ from the model. This modified model showed that as T1 CSE was lower and T1 loss was higher, the cusp (i.e., bifurcation) in distress occurred between T1 and T3 (Table 2). Female gender was not significant, β = −0.02, *p* = 0.777. Time since the wildfire was not significant, β = 0.02, *p* = 0.601. Thus, the final model comprised *z*^2^, T1 CSE, and T1 loss. AIC and BIC for the final model were 123.34 and 137.40, respectively, and adjusted *R*^2^ was 0.11.

#### Linear Model

To compare between the cusp model and a linear model, we ran a linear regression with the residualized scores between T1 distress and T3 distress. Independent variables included T1 CSE and T1 loss. Results showed that T1 CSE, β = −0.03, *p* = 0.463, and T1 loss, β = 0.07, *p* = 0.111, were not significant. AIC and BIC for this model were 133.23 and 144.48, and adjusted *R*^2^ was 0.02.

### Discussion

Results of study 1 showed that the upward non-linear shift in distress occurred between the initial assessment (2 months) and the 3-month assessment (4 months). CSE measured at the initial assessment was a bifurcation factor affecting when the cusp in distress occurred. The lower CSE scores are, the more the cusp in distress from the lower state to the upper state is likely to occur. In addition, perceived loss due to the wildfire was a significant asymmetry controlling factor affecting the strength of each state. The higher perceived loss scores are, the stronger the likelihood of being in the higher plane (or state) of distress. This non-linear cusp model explains the data better than the linear model based on the comparison of AICs.

The cusp in distress between the initial assessment and the 1-month follow-up may have occurred, but CSE and perceived loss were not identified as a bifurcation factor and an asymmetry controlling factor, respectively. Similarly, the cusp model for distress between time 2 (1-month follow-up) and 3-month assessment was not supported. This model indicates that the cusp did not occur during this period and CSE and loss were not a bifurcation factor or an asymmetry controlling factor, respectively. Thus, the non-linear dynamic shift was only seen over the entire 3-month wildfire recovery period and not within these shorter time periods. The overall time elapsed since the wildfire may be a key factor for a cusp to occur, although it was not identified as an asymmetry controlling factor. Previous longitudinal disaster research has typically demonstrated a linear reduction in psychological distress over time, yet several disaster recovery studies have also found no effect for time (Norris et al., 2001). In study 2, we attempted to replicate these findings in a sample of California wildfire survivors.

## Study 2: California Wildfires 2017–2018

Study 2 involved survivors who were affected by one of a series of California wildfires (Thomas Fire, Holiday Fire, Carr Fire, Camp Fire, and Woolsey Fire) between 2017 and 2018. Participants were residents who were directly affected by the fire. Based on the findings from study 1 that showed the importance of CSE as a critical bifurcation variable, we hypothesized that CSE perceptions would serve as a key catalyst or bifurcation factor in predicting a non-linear shift. For the asymmetry controlling factors, Study 1 findings suggested that damage/loss associated with the wildfire was important as an asymmetry controlling factor affecting the strength of the different states. In addition, we also assessed peritraumatic dissociation due to our previous MVA study (Benight et al., 2017) where peritraumatic dissociation was identified as an important asymmetry controlling factor. Finally, even though time since the disaster was not found in Study 1 to be an important asymmetry controlling variable, we included it in these cusp analyses due to the different study 2 sampling time frame.

### Method

#### Participants

A total of 148 people [*M*_*age*_ = 42.96 (*SD* = 14.39), 72.9% female] who suffered from a wildfire that occurred during 2017 and 2018 took part in the initial assessment (T1). The wildfires they experienced included the Thomas Fire (56.6%), the Holiday Fire (10.5%), the Carr Fire (6.3%), the Camp Fire (25.9%), and Woolsey Fire (0.7%). Among these 148 people, 82 of them completed the 6-week assessment (T2), and 66 of them finished the 6-month assessment (T3). The mean time elapsed since the wildfire was 287.73 days (*SD* = 130.99) at the time of the initial assessment. Participants were eligible for the study if they (a) owned an updated smartphone (<5 years old), (b) could speak and respond to questions in English, and (c) lived in the wildfire disaster-affected neighborhood (within three blocks of damaged or destroyed homes), or experienced property damage, or knew someone who was injured or had died as a result of the fire.

Most participants were well-educated with over two-thirds having at least some college up to a graduate degree. In kind, most of the sample also reported middle to upper middle socio-economic levels with annual income of more than $55,000 per year [$55,000–$85,000 (18.9%), greater than $85,000 (35.0%)]. Participants reported that marital status included never married (19.3%), married and living together (45.5%), married but living apart (5.5%), married but previously divorced (1.4%), living with a partner (12.4%), widowed (1.4%), or divorced (14.5%). Finally, the vast majority of the participants reported Caucasian as their ethnicity (82.4%), with the remaining 17.6% split among Hispanic (10.1%), African American (6.1%), Asian (0.7%), Native American/Alaskan (1.4%), or other (5.4%).

#### Measures

The measures for this specific analysis included posttraumatic distress, CSE for trauma, and peritraumatic dissociation at all three time points.

##### Distress

The Impact of Events Scale-Revised 6 (IES-6; Thoresen et al., 2010) was used to measure posttraumatic distress. The IES-Revised 6 is a six-item measure that assesses the presence and severity of posttraumatic distress. This scale is adapted from the longer 22-item version Impact of Events Scale-Revised (IES-R; Weiss, 2004). The scale measures intrusions (criteria B), avoidance (criteria C), and hyperarousal (criteria D). Respondents rate the severity of an item using a five-point scale ranging from 0 (*not at all*) to 4 (*extremely*). The IES-6 has demonstrated good internal consistency and convergent validity (Thoresen et al., 2010). Cronbach’s alpha coefficients for the present study were 0.91 at T1, 0.93 at T2, and 0.94 at T3.

##### Coping self-efficacy

Trauma Coping Self-Efficacy Scale (CSE-T; Benight et al., 2015) was used to measure perceived capability to deal with uncertainty and challenges associated with the wildfire. The CSE-T comprises nine items with a seven-point scale ranging from 1 (*not at all capable*) to 7 (*very capable*). Sample items included “Not ‘lose it’ emotionally” and “get my life back to normal.” The internal consistency (α = 0.93), test–retest reliability, and convergent validity of this scale have been validated with three separate samples, including disaster survivors (Benight et al., 2015). For the current study, Cronbach’s alpha coefficients were 0.91 at T1, 0.95 at T2, and 0.96 at T3.

##### Disaster exposure (damage/loss)

Exposure levels to the wildfires were assessed using 11 items developed for the current study. Respondents answered these items with a yes-or-no format. Items included “Did you see flames nearby?”, “Did you see smoke?”, “Did you have to protect against inhaling smoke (e.g., wear a face mask, keep doors/windows shut)?”, “Did you get sick or hurt during the wildfire?”, “Did you have to move temporarily at any point?”, “Were you seriously injured?”, “At any moment were you afraid of dying or getting injured?”, “Was someone close to you seriously injured?”, “Did you lose your place of residence because of the fire?”, “Was your neighborhood affected by the wildfire (e.g., homes in your neighborhood damaged/destroyed)?”, and “Did the wildfire damage/destroy items of sentimental, emotional, value such as family pictures, documents, trophies or other memorabilia?” Total scores were computed by summing the responses for “yes.” Cronbach’s alpha coefficient was relatively low (α = 0.55), indicating that participants had variety of different exposures to the wildfires.

##### Peritraumatic dissociation

The Peritraumatic Dissociative Experiences Questionnaire (PDEQ; Marmar et al., 2004) is a 10-item measure that was used to assess participant perceptions of dissociation during or immediately following the natural disaster. For each item, participants report the extent of their dissociation on a six-point scale ranging from 0 (*not at* all) to 5 (*extremely* true). Sample items included “My sense of time changed – things seemed to be happening in slow motion” and “I felt disoriented; that is, there were moments when I felt uncertain about where I was or what time it was.” Marmar et al. (2004) demonstrated that the PDEQ has good internal consistency, convergent validity, divergent validity, and predictive validity. Cronbach’s alpha coefficient was 0.92 at T1.

##### Demographics

Participants completed a demographic questionnaire at baseline that inquired about characteristics such as gender, ethnicity, age, socio-economic status, education, and time since the fire.

#### Procedures

Participants were contacted via several recruitment methods (e.g., social media, newspaper advertisement, flyers, tabling within affected communities, and phone calls to target zip codes) about study enrollment. Those who expressed interest following these recruitment methods were then screened for study eligibility. Eligible participants were guided through the informed consent process that included having the participants select which individual or combination of study aspects they wanted to take part (online surveys, daily app surveys, and sensor data collection). After providing consent, they completed a baseline survey online. Participants completed 6-week and 6-month follow-up surveys. They were compensated $20 for completing each survey and provided with a list of community resources (e.g., local mental health clinics, disaster relief organizations) at each time point.

#### Data Analysis

Parallel to Study 1, cusp catastrophe models were performed using a polynomial regression analysis on residualized scores of distress. Three different polynomial regressions were performed based on the different time frames (T1 with T2, T1 with T3, and T2 with T3). Independent variables included *z*^3^, *z*^2^, beta (T1 CSE × *z*) as a bifurcation factor, and disaster exposure, history of traumatic life events, time since the wildfire, gender, and peritraumatic dissociation as asymmetry controlling factors. Again, a linear regression was run as a comparison.

#### Missing Data

After excluding respondents who did not complete any of the items from the study measures, 0.32% at T1 and 0.12% at T3 were missing data. There were no missing data at T2. These missing data were imputed using an inverse non-linear principal component analysis method using an R package “pcaMethods” (Stacklies et al., 2007).

### Results

#### Attrition Analysis

Attrition analyses showed that there were no significant differences between completers of T2 and dropouts of T2 in T1 distress, T1 CSE, peritraumatic dissociation, age, annual income, education levels, disaster exposure, and time since the wildfire, *t* range = 0.09–1.68, *p* range = 0.095–0.925. These groups were also not significantly different based on gender, χ^2^(2) = 3.75, *p* = 0.153. Results of attrition analyses between T3 completers and T3 dropouts showed that there were no differences in T2 distress, CSE, age, education levels, annual income, disaster exposure, and time since the wildfire, *t* range = 0.02–1.49, *p* range = 0.139–0.985. There was also no difference based on gender between T3 completers and T3 dropouts, χ^2^(2) = 3.83, *p* = 0.148.

#### Preliminary Findings

Results of Pearson’s correlations showed that there were negative associations with medium to large effects between distress and CSE across all time points (Table 3). Distress across all time points had positive associations with T1 peritraumatic dissociation with medium to large effect and had positive associations with traumatic life events with medium to large effect. There were negative associations between CSE across all time points and T1 peritraumatic dissociation with a medium effect and traumatic life events with small to medium effect. Peritraumatic dissociation and traumatic life events had a positive association with a medium effect. Time since the wildfire had negative and small to medium effects with distress and peritraumatic dissociation. It had a positive and small effect with T2 CSE. Finally, disaster exposure positively related to distress, peritraumatic dissociation, and traumatic life event with small to medium effects. Disaster exposure and CSE were negatively related with small to medium effects.

**TABLE 3:** Pearson’s correlations, means, and standard deviations for the study variables for Study 2.

	**1**	**2**	**3**	**4**	**5**	**6**	**7**	**8**	**9**
(1) T1 distress									
(2) T2 distress	0.66***								
(3) T3 distress	0.63***	0.72***							
(4) T1 CSE	−0.56***	−0.50***	−0.60***						
(5) T2 CSE	−0.60***	−0.77***	−0.75***	0.65***					
(6) T3 CSE	−0.48***	−0.58***	−0.78***	0.50***	0.64***				
(7) T1 PD	0.59***	0.49***	0.61***	−0.50***	−0.49***	−0.46***			
(8) Time since	−0.33***	−0.28*	−0.24*	0.06	0.22*	0.17	−0.17*		
(9) Exposure	0.42***	0.41***	0.26*	−0.43***	−0.33**	–0.07	0.24	–0.07	
Mean	1.93	1.37	1.32	5.07	5.23	5.13	2.43	287.7	6.75
*SD*	1.08	1.10	1.13	1.22	1.45	1.54	1.01	130.99	1.79

***p < 0.001, **p < 0.01, *p < 0.05. T1, time 1; T2, time 2; T3, time 3; CSE, coping self-efficacy; Exposure, disaster exposure; PD, peritraumatic dissociation; SD, standard deviation.

#### Polynomial Catastrophe Cusp

Three different time frames were used for testing non-linear dynamics. Polynomial regressions for each time frame are reported below.

##### Time 1 to time 2 cusp

We ran a polynomial regression analysis on residualized scores between T1 distress and T2 distress as a dependent variable, T1 CSE (T1 CSE × *z*) as a bifurcation factor, disaster exposure, gender, and time since the wildfire as asymmetry controlling factors, and *z*^3^ and *z*^2^ (*n* = 78). Results showed that *z*^3^ and *z*^2^ were not significant; thus, *z*^3^ was removed to modify the model. Results of the modified model showed that *z*^2^ was still not significant (Table 4). These results indicated that the cusp in distress did not occur between T1 and T2. AIC and BIC for this model were 195.86 and 212.27, respectively, and adjusted *R*^2^ was 0.05.

**TABLE 4:** Standardized coefficients, standard error, and *p*-values for the cusp model in distress between time 1 and time 2 and in distress between time 1 and time 3 for Study 2.

**DV**	**IV**	**β**	**Std. error**	***p***
****Δ** distress T1–T2**				
	*z*^2^	0.11	0.16	0.463
	T1 CSE × *z*	–0.33	0.15	0.036
	Disaster exposure	0.19	0.10	0.071
	Time since	–0.16	0.11	0.134
	Female gender	0.02	0.24	0.917
****Δ** distress T1–T2**				
	*z*^2^	–0.04	0.18	0.814
	T1 CSE × *z*	–0.17	0.15	0.278
	PD	0.21	0.11	0.064
	Time since	–0.16	0.10	0.138
	Female gender	–0.00	0.23	0.990
****Δ** distress T1–T3**				
	*z*^3^	–1.83	0.83	0.032
	*z*^2^	2.37	0.93	0.014
	T1 CSE × *z*	–0.68	0.20	0.001
	Disaster exposure	–0.06	0.13	0.617
	Time since	–0.16	0.11	0.143
	Female gender	0.01	0.28	0.977
****Δ** distress T1–T3**				
	*z*^2^	–0.06	0.19	0.758
	T1 CSE × *z*	–0.20	0.16	0.204
	Peritraumatic dissociation	0.49	0.11	< 0.001
	Time since	–0.22	0.10	0.029
	Female gender	–0.02	0.24	0.928

*DV, dependent variable; IV, independent variable; β, standardized coefficient; T1, time 1; T2, time 2; T3, time 3; Std. error, standard error; CSE, coping self-efficacy.*

Next, we switched disaster exposure to peritraumatic dissociation as an asymmetry controlling factor in the same analysis. Results showed that *z*^3^ and *z*^2^ were approaching significance; thus, *z*^3^ was excluded from the model. The modified model showed that peritraumatic dissociation was the only significant variable, indicating that the cusp in distress did not occur between T1 and T2. AIC and BIC for this model were 198.55 and 215.14, respectively, and adjusted *R*^2^ was 0.05.

##### Time 1 to time 3 cusp

The second polynomial regression analysis included residualized scores between T1 distress and T3 distress (*n* = 62). The analysis included T1 CSE (T1 CSE × *z*) as a bifurcation factor and disaster exposure, gender, and time since the wildfires as asymmetry controlling factors. *z*^3^ and *z*^2^ were also included in the model. Results showed that *z*^3^, *z*^2^, and T1 CSE were significant (Table 4). These results indicated that the cusp in distress may have occurred, but the model was not supported because of non-significant asymmetry controlling factors. AIC and BIC for this model were 158.47 and 175.35, respectively, and adjusted *R*^2^ was 0.13.

A separate polynomial regression with peritraumatic dissociation as an asymmetry controlling factor was conducted (instead of disaster exposure) (*n* = 64). Results showed that *z*^3^ and *z*^2^ were not significant; thus, we removed *z*^3^ to modify the model. Results of the modified model showed that *z*^2^ was still not significant, indicating that the model was not supported. AIC and BIC for the modified model were 147.67 and 162.67, respectively, and adjusted *R*^2^ was 0.31.

##### Time 2 to time 3 cusp

We conducted a polynomial regression analysis on residualized scores between T2 distress and T3 distress (*n* = 62). This analysis included T2 CSE (T2 CSE × *z*) as a bifurcation factor and disaster exposure, gender, and time since the wildfire as asymmetry controlling factors. *z*^3^ and *z*^2^ were also included in the model. Results showed that *z*^3^, *z*^2^, and T2 CSE were significant (Table 5). These results suggested that the cusp in distress may have occurred between T2 and T3 with T2 CSE as a bifurcation factor, but the model was not supported because of non-significant asymmetry controlling factors. AIC and BIC were 133.90 and 150.79, respectively, and adjusted *R*^2^ was 0.24.

**TABLE 5:** Standardized coefficients, standard error, and *p*-values for the cusp model in distress between time 2 and time 3 for Study 2.

**DV**	**IV**	**β**	**Std. error**	***p***
****Δ** distress T2–T3**				
	*z*^3^	–3.07	0.75	< 0.001
	*z*^2^	3.50	0.82	< 0.001
	T2 CSE × *z*	–0.79	0.18	< 0.001
	Disaster exposure	0.03	0.10	0.735
	Time since	0.01	0.09	0.909
	Female gender	–0.06	0.23	0.783
****Δ** distress T2–T3**				
	*z*^3^	–2.25	0.67	0.001
	*z*^2^	2.47	0.75	0.002
	T2 CSE × *z*	–0.62	0.16	< 0.001
	Peritraumatic dissociation	0.31	0.09	0.002
	Time since	–0.05	0.09	0.597
	Female gender	–0.09	0.20	0.647

*DV, dependent variable; IV, independent variable; T1, time 1; T2, time 2; T3, time 3; β, standardized coefficient; Std. error, standard error; CSE, coping self-efficacy.*

Finally, the same analysis with peritraumatic dissociation as an asymmetry controlling factor (*n* = 62) showed that *z*^3^, *z*^2^, T2 CSE, and peritraumatic dissociation were all significant. Gender and time since the wildfire were not significant. This finding suggested that the cusp in distress occurred when T2 CSE decreased and peritraumatic dissociation increased. AIC and BIC for the final model was 124.77 and 141.91, respectively, explaining 38% of the variance (*R*^2^_*adj*_ = 0.38).

#### Linear Model

We conducted a linear regression analysis on the residualized scores between T2 distress and T3 distress to compare to our final cusp catastrophe model (*n* = 62). This analysis included T1 CSE, T2 CSE, peritraumatic dissociation, gender, and time since the wildfire as predictors. Results showed that peritraumatic dissociation was a significant predictor, β = 0.31, *p* = 0.003. T2 CSE, β = −0.14, *p* = 0.179, female gender, β = −0.17, *p* = 0.446, and time since the wildfire, β = −0.08, *p* = 0.379, were not significant. AIC and BIC for the linear model were 139.00 and 151.85, respectively, and adjusted *R*^2^ was 0.20.

#### Follow-Up Analysis With Camp Fire vs. Other Fires

There were some differences in the study variables between participants suffering from the Camp Fire and other fires. Comparison between these participants showed that values of time since the wildfire, disaster exposure, T1 distress, T1 CSE, and peritraumatic dissociation were significantly different (Table 6). Because of these differences, we excluded people suffering from the Camp Fire and conducted the same analyses (*n* = 106). Results of the polynomial regression analyses were consistent with the findings from the sample with participants suffering from the Camp Fire (see Tables 7, 8 for standardized coefficients for the cusp model excluding participants suffering from the Camp Fire).

**TABLE 6:** Camp fire and combined fire group baseline means and standard deviations for the study variables.

	**Camp fire**		**Other fires**		
**Variable**	**Mean**	***SD***	**Mean**	***SD***	***F***
Time since the wildfire	149.54	27.23	335.97	117.77	90.62
Disaster exposure	7.76	1.19	6.37	1.86	17.79
T1 distress	2.90	0.85	1.59	0.95	54.57
Peritraumatic dissociation	3.05	1.06	2.21	0.91	21.36
T1 CSE	4.20	1.21	5.37	1.09	29.94

*SD, standard deviation; T1, time 1; CSE, coping self-efficacy. F scores were calculated to compare the means between people who suffered from the Camp Fire and other fires combined. All means significantly differed, p < 0.001.*

**TABLE 7:** Standardized coefficients, standard error, and *p*-values for the cusp model in distress between time 1 and time 2 and in distress between time 1 and time 3 without participants suffering from the camp fire.

**DV**	**IV**	**β**	**Std. error**	***p***
****Δ** distress T1–T2**				
	*z*^2^	–0.08	0.25	0.755
	T1 CSE × *z*	–0.18	0.27	0.512
	Disaster exposure	0.06	0.14	0.674
	Time since	0.02	0.13	0.863
	Female gender	0.12	0.28	0.662
	AIC = 148.89; BIC = 162.94; *R*^2^_*adj*_ = −0.01			
****Δ** distress T1–T2**				
	*z*^2^	–0.32	0.24	0.177
	T1 CSE × *z*	0.04	0.23	0.853
	Peritraumatic dissociation	0.33	0.13	0.015
	Time since	–0.04	0.12	0.758
	Female gender	0.13	0.25	0.597
	AIC = 145.41; BIC = 159.71; *R*^2^_*adj*_ = 0.10			
****Δ** distress T1–T3**				
	*z*^2^	0.16	0.28	0.560
	T1 CSE × *z*	–0.34	0.29	0.248
	Disaster exposure	–0.11	0.16	0.493
	Time since	0.05	0.14	0.694
	Female gender	0.10	0.31	0.752
	AIC = 126.76; BIC = 139.57; *R*^2^_*adj*_ = -0.00			
****Δ** distress T1–T3**				
	*z*^2^	–0.19	0.23	0.429
	T1 CSE × *z*	–0.10	0.23	0.672
	Peritraumatic dissociation	0.54	0.12	< 0.001
	Time since	–0.12	0.11	0.279
	Female gender	0.08	0.25	0.753
	AIC = 115.23; BIC = 128.33; *R*^2^_*adj*_ = 0.29			

*DV, dependent variable; IV, independent variable; T1, time 1; T2, time 2; T3, time 3; β, standardized coefficient; Std. error, standard error; CSE, coping self-efficacy.*

**TABLE 8:** Standardized coefficients, standard error, and *p*-values for the cusp model in distress between time 2 and time 3 without participants suffering from the camp fire.

**DV**	**IV**	**β**	**Std. error**	***p***
****Δ** distress T2–T3**				
	*z*^3^	–2.45	0.74	0.002
	*z*^2^	2.85	0.85	0.002
	T2 CSE × *z*	–0.81	0.22	< 0.001
	Disaster exposure	0.04	0.13	0.779
	Time since	0.03	0.11	0.794
	Female gender	–0.03	0.25	0.912
	AIC = 108.14; BIC = 122.77; *R*^2^_*adj*_ = 0.19			
****Δ** distress T2–T3**				
	*z*^3^	–1.89	0.65	0.006
	*z*^2^	2.06	0.76	0.009
	T2 CSE × *z*	–0.64	0.18	0.001
	Peritraumatic dissociation	0.32	0.12	0.009
	Time since	–0.03	0.09	0.785
	Female gender	0.01	0.22	0.966
	AIC = 102.09; BIC = 117.06; *R*^2^_*adj*_ = 0.35			

*DV, dependent variable; IV, independent variable; T2, time 2; T3, time 3; β, standardized coefficient; Std. error, standard error; CSE, coping self-efficacy.*

### Discussion

Importantly, Study 2 findings provided confirmation of the non-linear dynamic shifts in recovery from wildfires. The results show that a distress cusp occurs between approximately 10.5 months and 1 year 4 months after the wildfire. It is also important to note the different time frame for this cusp effect in comparison to Study 1 where we identified the non-linear shift between the initial assessment (approximately 2 months) and 4 months after the fire. CSE measured at 10.5 months serves an important role in determining when the cusp occurs (bifurcation factor). Peritraumatic dissociation affects the strength of each state in the cusp (asymmetry controlling factor). The lower T2 CSE scores are, the more the cusp in distress is likely to occur from the lower distress state to the higher distress state. The higher peritraumatic dissociation scores are, the stronger the attractiveness of scores to be in the higher state of distress. Based on the comparison of AIC, the cusp model is better than the linear model indicated by a lower AIC and explaining a greater amount of the variance.

## General Discussion

Polynomial regression cusp catastrophe analyses confirmed the importance of CSE perceptions after the disasters as a bifurcation factor for non-linear shifts in distress during the early stages of recovery from approximately 2 months and 4 months in Study 1 and during more intermediate recovery for Study 2 (10.5 months to 1 year 4.5 months). In both cases, the bifurcation into a higher state of distress occurred as CSE levels dropped. In Study 1, the greater the loss from the fire, the more attractive the higher phase of distress becomes, creating ripe conditions for CSE to trigger a bifurcation of distress into a non-linear surge. For study 2, we did not see this effect with loss. We did, however, observe this with peritraumatic dissociation where higher peritraumatic dissociation created a vulnerability for the effect of dropping CSE, promoting a bifurcation of distress into a non-linear upward shift.

Perceptions of CSE appear to consistently play a pivotal role in promoting a negative shift in functioning. We identified this in the current samples and in our two previous MVA studies. This is consistent with and supportive of SRST. Self-regulation shift theory suggests that a major non-linear shift in functioning will occur when one’s CSE perceptions drop to the point where the survivor simply stops believing he/she can manage the demands (i.e., the self-determination violation effect).

The vulnerability factors for our wildfire samples (asymmetry controlling factors), however, differed from our MVA studies. In the MVA samples, we found that the MVA emergency room patients who had lower levels of either injury severity or peritraumatic dissociation were the ones who demonstrated a non-linear shift 3 months later. We speculated that these individuals would expect they would recovery easily. Yet, for those with lower levels of CSE, this was not the case, possibly setting up a self-regulatory mismatch (i.e., “I should be getting better, but I don’t think I can handle this”) that contributed to the non-linear negative shift in function.

In contrast, both wildfire samples showed that greater disaster exposure or peritraumatic dissociation set the stage for lower CSE to promote a non-linear upward shift (bifurcation) in distress. Thus, those who were more negatively affected by the wildfires were more at risk for the influence of negative CSE perceptions pushing the non-linear upward shift.

The discrepancy in these findings underscores the importance of trauma recovery context (MVA recovery vs. wildfire disaster) in non-linear dynamics related to recovery. MVA survivors, in this case those considered low risk, are challenged with significant external (e.g., legal complications and transportation difficulties) and internal (e.g., memories of the accident and fears of driving again) stressors. Wildfire recovery, in contrast, presents a very different set of stressors that are community wide and intensely personal. Large-scale wildfires can be perceived as cultural trauma that has profound consequences in people living in the targeted community (Alexander, 2004). People might experience a multitude of sufferings with cultural trauma that are qualitatively different from sufferings experienced in other types of trauma such as MVA, including geographical displacement, destruction of social networks, and loss of property. The landscape is drastically altered with reminders everywhere of the disaster. Many will have rebuilding challenges including working with insurance companies, finding contractors, etc. Thus, the recovery context for these two different potentially traumatic events is drastically different and may have influenced the contextual conditions through which CSE influences the self-regulation shift and non-linear increases in distress.

The results of these different trauma-exposed samples have important implications for basic science research. These findings provide more support for a key tenet of SRST. It suggests that humans have a critical threshold on the spectrum of CSE (self-determination violation point). Survivors hit a critical threshold when they perceive regaining a sense of control is impossible during their recovery. This point is where a non-linear shift occurs from a relatively healthy stable state to another impaired stable state (i.e., new equilibrium). More research over different time series denominations (e.g., days, weeks, months, or years) may provide a clearer look at the oscillations of non-linear state changes in trauma populations. One would expect possible positive shifts to occur as individuals regain a sense of control either through reductions in environmental challenges (e.g., community rebuilding) or through social support enabling of recovery skills (Benight et al., 2018).

A polynomial regression analysis for cusp catastrophe model provides evidence of a non-linear shift in distress from one state to another state although whether these states are stable is unclear in this analytical approach. Previous studies have shown that CSE plays a bifurcation role for condom-use intention (Yu et al., 2018) and post-treatment alcohol use (Witkiewitz et al., 2007). These previous studies and our findings underscore the importance of self-evaluative judgments (i.e., CSE) in non-linear dynamics.

It is also important to investigate critical asymmetry controlling factors that, when paired with a bifurcation variable, push individuals toward a non-linear shift in state. Specific asymmetry variables need to be hypothesized relative to particular contexts. Within the disaster recovery environment, for example, our findings suggest that people tend to be in the impaired state more often when perceived loss or peritraumatic dissociation is higher. These factors are important in other linear-based studies on disaster recovery (Norris et al., 2002; Ozer et al., 2008). Other potential asymmetry controlling factors within disaster recovery include, but are not limited to, significant life threat, serious injury, exposure to human remains, low perceived social support, significant post-disaster stressors, female gender, previous psychiatric status, and minority status (Norris et al., 2002). The effects of these variables on non-linear dynamics in posttraumatic distress may vary by the type of disaster (man-made versus natural), time since the disaster, or the type of sample (e.g., seeking mental health services versus community; Ozer et al., 2008). Thus, a cusp model needs to include suitable asymmetry controlling factors for particular samples.

The results of our two wildfire disaster recovery studies with different time frames suggest that a non-linear shift may occur over different time trajectories. We found that the upward shift in distress occurs when a time gap is greater than 2 months, although the models with a shorter period between two time points were not supported. A previous study indicated that a time gap between two time points may need to be long enough for a cusp to occur as well. Further studies will need to confirm the exact duration that is needed for a cusp to be observed.

### Intervention Implications

The present studies have some intervention implications. Our studies indicate that CSE, perceived loss, and peritraumatic dissociation play important roles in the negative non-linear shift in distress. Although the extent of loss is a difficult variable to influence after a disaster, it may be possible through resource replenishment that the perception of loss can be improved. In addition, the importance of self-appraisals of CSE can be targeted directly with post-disaster interventions. For example, CSE can be enhanced, assisting survivors in specific goal attainment in the recovery process to enhance perceptions of mastery (e.g., getting an insurance agent to come to the property or finding a contractor to help with rebuilding). Social modeling of effective coping and recovery strategies and supportive persuasion from friends and response personnel can help to promote reappraisals of traumatic experiences and positive interpretation of one’s coping capabilities (Ozer and Bandura, 1990; Rothbaum et al., 2001; Rothbaum and Schwartz, 2002; Benight and Bandura, 2004).

### Limitations

There are important limitations to mention when interpreting these two studies. First, the posttraumatic distress measure for Study 1 did not include hyperarousal symptoms. This may have influenced the construct validity of this measure of distress. However, Study 2 provides some reassurance in that the results were consistent with Study 1. It should be noted, however, that the measure for distress differed between the two studies. Thus, future investigations are necessary to confirm non-linear shifts in distress predicted by SRST that utilize contemporary posttraumatic stress measurement (e.g., the PCL-5).

Second, the analytic method utilized in both studies does not provide a way to assess the stability of states identified in both samples. To evaluate if these states are stable, a different analytical method such as Markov regime switching model would be useful. Self-regulation shift theory predicts that a critical self-determination threshold where CSE perceptions are too low to manage environmental challenges results in these non-linear shifts in distress. What the theory does not include is the possibility for oscillations between states and the impact of this on coping effectiveness and long-term distress. Many psychological factors including distress fluctuate daily (Kukk and Akkermann, 2017; Pihet et al., 2017; Maher et al., 2019). Thus, future studies that utilize different non-linear analytic techniques are needed to refine the multiple shift question in trauma adaptation.

Third, because these two studies were drawn from larger studies with different aims, we did not have the exact same variables across the two studies. However, the key bifurcation variable (CSE) was included in both and was found to be a critical bifurcation variable as predicted by SRST. The two studies share similar variables, perceived loss, and disaster exposure, yet only Study 2 measured peritraumatic dissociation. Interestingly, loss was found to be an important asymmetry controlling factor in Study 1, but peritraumatic dissociation in Study 2. This difference raises important conceptual and practical questions regarding the disaster context and the individual psychological experience during the disaster in creating the drive toward one state or another as recovery unfolds.

Lastly, the generalization beyond these two select samples is limited. Disaster research has varied in finding selection biases with some reporting higher level of psychological problems participating more (Grievink et al., 2006), less (Ginexi et al., 2000), or stayed the same (Norris et al., 2001). Without pre-data on the disaster participants, it therefore remains unknown if our sample was truly biased relative to those who did not choose to participate. Specific to study 2, significant attrition also calls into question a sample bias that needs to be considered. It remains unknown what factors differentiate those who continued in the study versus those who dropped out. Together, these issues suggest caution in the interpretation of the present findings.

## Conclusion

The findings from the two wildfire recovery samples offer further support for the theoretical prediction from SRST that CSE perceptions play a pivotal role in a self-determination threshold leading to a non-linear shift in distress states. We found that a cusp in distress occurs in the aftermath of wildfires in two separate wildfire samples: survivors of the Waldo Canyon wildfire in Colorado and those of California wildfires occurring between 2017 and 2018. As CSE decreases, an upward shift in distress is more likely to occur. Furthermore, these two studies showed that perceived loss and peritraumatic dissociation are important asymmetry controlling factors. These findings support the tenets of SRST. As these variables become higher, distress is more likely to be on the higher plane. These two studies had very different time frames raising important questions relative to non-linear dynamic shifts during the wildfire recovery process. Future research methodologies that specifically target different time frames will help to address this issue. Although there are several limitations in the present studies, these studies provided the first evidence of a non-linear shift in distress aftermath of wildfires and provided support for SRST.

## Data Availability Statement

The raw data supporting the conclusions of this article will be made available by the authors, without undue reservation, to any qualified researcher.

## Ethics Statement

The studies involving human participants were reviewed and approved by Institutional Review Board at the University of Colorado Colorado Springs and Institutional Review Board at the University of California, Santa Barbara. The patients/participants provided their written informed consent to participate in this study.

## Author Contributions

CB was responsible for the design and conception of the studies, writing the initial draft, revising the manuscript, and disseminating the findings. KS worked on the data analysis, the initial draft, and revising the manuscript. AH was responsible for data management and writing the initial draft. EF conducted study 2, collected the data, and revised the manuscript. All authors contributed to the article and approved the submitted version.

## Conflict of Interest

The authors declare that the research was conducted in the absence of any commercial or financial relationships that could be construed as a potential conflict of interest.

## References

[B1] Alexander J. (2004). “Toward a theory of cultural trauma,” in *Cultural Trauma and Collective Identity*, ed. Alexander J. (Berkeley, CA: University of California Press), 1–30. 10.1525/california/9780520235946.003.0001

[B2] Bandura A. (1997). *Self-Efficacy: The Exercise of Control.* New York, NY: Henry Holt.

[B3] Benight C. C. Bandura A. (2004). Social cognitive theory of posttraumatic recovery: the role of perceived self-efficacy. *Behav. Res. Ther.* 42 1129–1148. 10.1016/j.brat.2003.08.008 15350854

[B4] Benight C. C. Harper M. L. (2002). Coping self-efficacy perceptions as a mediator between acute stress response and long-term distress following natural disasters. *J. Trauma Stress* 15 177–186. 10.1023/A:1015295025950 12092909

[B5] Benight C. C. Harwell A. Shoji K. (2018). Self-regulation shift theory: a dynamic personal agency approach to recovery capital and methodological suggestions. *Front. Psychol.* 9:1738. 10.3389/fpsyg.2018.01738 30298033 PMC6160534

[B6] Benight C. C. McFarlane A. C. (2007). Challenges for disaster research: recommendations for planning and implementing disaster mental health studies. *J. Loss Trauma* 12 419–434. 10.1080/15325020701285128

[B7] Benight C. C. Shoji K. Delahanty D. L. (2017). Self-regulation shift theory: a dynamic systems approach to traumatic stress. *J. Trauma Stress* 30 333–342. 10.1002/jts.22208 28741845

[B8] Benight C. C. Shoji K. James L. E. Waldrep E. E. Delahanty D. L. Cieslak R. (2015). Trauma coping self-efficacy: a context-specific self-efficacy measure for traumatic stress. *Psychol. Trauma* 7 591–599. 10.1037/tra0000045 26524542 PMC4664052

[B9] Benight C. C. Swift E. Sanger J. Smith A. Zeppelin D. (1999). Coping self-efficacy as a mediator of distress following a natural disaster. *J. Appl. Soc. Psychol.* 29 2443–2464. 10.1111/j.1559-1816.1999.tb00120

[B10] Bosmans M. W. G. Benight C. C. van der Knaap L. M. Winkel F. W. van der Velden P. G. (2013). The associations between coping self-efficacy and posttraumatic stress symptoms 10 years postdisaster: differences between men and women. *J. Trauma Stress* 26 184–191. 10.1002/jts.21789 23526650

[B11] Burnham K. P. Anderson D. R. (2004). Multimodel inference: understanding AIC and BIC in model selection. *Sociol. Methods Res.* 33 261–304. 10.1177/0049124104268644

[B12] Burnham K. P. Anderson D. R. Huyvaert K. P. (2011). AIC model selection and multimodel inference in behavioral ecology: some background, observations, and comparisons. *Behav. Ecol. Sociobiol.* 65 23–35. 10.1007/s00265-010-1029-6

[B13] California Department of Forestry and Fire Protection (2019a). *Camp Fire.* Available online at: https://www.fire.ca.gov/incidents/2018/11/8/camp-fire/ (accessed December 1, 2019).

[B14] California Department of Forestry and Fire Protection (2019b). *Carr Fire.* Available online at: https://www.fire.ca.gov/incidents/2018/7/23/carr-fire/ (accessed December 1, 2019).

[B15] California Department of Forestry and Fire Protection (2019c). *Holiday Fire.* Available online at: https://www.fire.ca.gov/incidents/2018/7/6/holiday-fire/ (accessed December 1, 2019).

[B16] California Department of Forestry and Fire Protection (2019d). *Woolsey Fire.* Available online at: https://www.fire.ca.gov/incidents/2018/11/8/woolsey-fire/ (accessed December 1, 2019).

[B17] Deci E. L. Ryan R. M. (2000). The “what” and “why” of goal pursuits: human needs and the self-determination of behavior. *Psychol. Inq.* 11 227–268. 10.1207/S15327965PLI1104_01

[B18] Denver Post (2012). *Colorado Wildfire: Waldo Canyon Fire Destroyed 346 Homes, 1 Dead, 1 Missing.* Denver: The Denver Post.

[B19] Finkelstein M. (2016). Resource loss, resource gain, PTSD, and dissociation among Ethiopian immigrants in Israel. *Scand. J. Psychol.* 57 328–337. 10.1111/sjop.12295 27291081

[B20] Ford D. H. (1987). *Humans as Self-Constructing Living Systems: A Developmental Perspective on Behavior and Personality.* New Jersey: Lawrence Erlbaum Associates.

[B21] Freedy J. R. Saladin M. E. Kilpatrick D. G. Resnick H. S. Saunders B. E. (1994). Understanding acute psychological distress following natural disaster. *J. Trauma Stress* 7 257–273. 10.1002/jts.2490070207 8012746

[B22] Galea S. Nandi A. Vlahov D. (2005). The epidemiology of post-traumatic stress disorder after disasters. *Epidemiol. Rev.* 27 78–91. 10.1093/epirev/mxi003 15958429

[B23] Gill M. A. Stephens S. L. Cary G. J. (2013). The worldwide “wildfire” problem. *Ecol. Appl.* 23 438–454. 10.1890/10-2213.1 23634593

[B24] Ginexi E. M. Weihs K. Simmens S. J. Hoyt D. R. (2000). Natural disaster and depression: a prospective investigation of reactions to the 1993 midwest floods. *Am. J. Commun. Psychol.* 28 495–518. 10.1023/A:1005188515149 10965388

[B25] Grasman R. P. P. P. van der Maas H. L. J. Wagenmakers E.-J. (2009). Fitting the cusp catastrophe in R: a cusp package primer. *J. Stat. Softw.* 32 1–27. 10.18637/jss.v032.i08

[B26] Green B. L. (1998). Psychological responses to disasters: conceptualization and identification of high-risk survivors. *Psychiatry Clin. Neurosci.* 52 S25–S31. 10.1046/j.1440-1819.1998.0520s5s67.x

[B27] Grievink L. Van der Velden P. G. Yzermans C. J. Roorda J. Stellato R. K. (2006). The importance of estimating selection bias on prevalence estimates shortly after a disaster. *Ann. Epidemiol.* 16 782–788. 10.1016/j.annepidem.2006.04.008 16882468

[B28] Guastello S. J. (1982). Moderator regression and the cusp catastrophe: application of two-stage personnel selection, training, therapy, and policy evaluation. *Behav. Sci.* 27 259–272. 10.1002/bs.3830270305

[B29] Guastello S. J. (1987). A butterfly catastrophe model of motivation in organization: academic performance. *J. Appl. Psychol.* 72 165–182. 10.1037/0021-9010.72.1.165

[B30] Guerra C. Cumsille P. Martínez M. L. (2014). Post-traumatic stress symptoms in adolescents exposed to an earthquake: association with self-efficacy, perceived magnitude, and fear. *Int. J. Clin. Health Psychol.* 14 202–207. 10.1016/j.ijchp.2014.05.001

[B31] Hirschel M. J. Schulenberg S. E. (2009). Hurricane Katrina’s impact on the Mississippi Gulf coast: general self-efficacy’s relationship to PTSD prevalence and severity. *Psychol. Serv.* 6 293–303. 10.1037/a0017467

[B32] Hobfoll S. E. (1989). Conservation of resources: a new attempt at conceptualizing stress. *Am. Psychol.* 44 513–524. 10.1037/0003-066X.44.3.513 2648906

[B33] Hobfoll S. E. (1991). Traumatic stress: a theory based on rapid loss of resources. *Anxiety Res.* 4 187–197. 10.1080/08917779108248773

[B34] Kass R. E. Raftery A. E. (1995). Bayes factors. *J. Am. Stat. Assoc.* 90 773–795. 10.1080/01621459.1995.10476572

[B35] Kukk K. Akkermann K. (2017). Fluctuations in negative emotions predict binge eating both in women and men: an experience sampling study. *Eat. Disord.* 25 65–79. 10.1080/10640266.2016.1241058 27775488

[B36] Linton E. (2012). *Colorado Springs Fire Toll: 346 Homes, No Injuries.* Manhattan, NY: International Business Times.

[B37] Luszczynska A. Benight C. C. Cieslak R. (2009). Self-efficacy and health-related outcomes of collective trauma: a systematic review. *Eur. Psychol.* 14 51–62. 10.1027/1016-9040.14.1.51

[B38] Maher J. P. Dzubur E. Nordgren R. Huh J. Chou C.-P. Hedeker D. (2019). Do fluctuations in positive affective and physical feeling states predict physical activity and sedentary time? *Psychol. Sport Exerc.* 41 153–161. 10.1016/j.psychsport.2018.01.011 30853854 PMC6402603

[B39] Marmar C. R. Metzler T. J. Otte C. (2004). “The Peritraumatic Dissociative Experiences Questionnaire,” in *Assessing Psychological Trauma and PTSD*, 2nd Edn, eds Wilson J. P. Keane T. M. (New York, NY: Guilford Press), 144–167.

[B40] Marshall G. N. Schell T. L. Elliott M. N. Rayburn N. R. Jaycox L. H. (2007). Psychiatric disorders among aults seeking emergency disaster assistance after a wildland-urban interface fire. *Psychiatr. Serv.* 58 509–514. 10.1176/ps.2007.58.4.509 17412853

[B41] National Interagency Fire Center (2018). *Total Wildland Fires and Acres (1926-2018) [Total wildland fires and acres (1926-2018)].* Boise: National Interagency Fire Center.

[B42] Neria Y. Nandi A. Galea S. (2008). Post-traumatic stress disorder following disasters: a systematic review. *Psychol. Med.* 38 467–480. 10.1017/S0033291707001353 17803838 PMC4877688

[B43] Norris F. H. (2005). *Range, Magnitude, and Duration of the Effects of Disasters on Mental Health: Review Update 2005.* Hanover, NH: National Center for PTSD.

[B44] Norris F. H. Friedman M. J. Watson P. J. (2002). 60,000 disaster victims speak: part II. Summary and implications of the disaster mental health research. *Psychiatry* 65:240. 10.1521/psyc.65.3.207.20173 12405080

[B45] Norris F. H. Murphy A. D. Kaniasty K. Perilla J. L. Ortis D. C. (2001). Postdisaster social support in the United States and Mexico: conceptual and contextual considerations. *Hispa. J. Behav. Sci.* 23 469–497. 10.1177/0739986301234008

[B46] North C. S. Oliver J. Pandya A. (2012). Examining a comprehensive model of disaster-related posttraumatic stress disorder in systematically studied survivors of 10 disasters. *Am. J. Public Health* 102 e40–e48. 10.2105/AJPH.2012.300689 22897543 PMC3490647

[B47] North C. S. Pfefferbaum B. (2013). Mental health response to community disasters: a systematic review. *JAMA* 310 507–518. 10.1001/jama.2013.107799 23925621

[B48] Ozer E. J. Best S. R. Lipsey T. L. Weiss D. S. (2008). Predictors of posttraumatic stress disorder and symptoms in adults: a meta-analysis. *Psychol. Trauma* 129 52–73. 10.1037/1942-9681.S.1.3 12555794

[B49] Ozer E. M. Bandura A. (1990). Mechanisms governing empowerment effects: a self-efficacy analysis. *J. Pers. Soc. Psychol.* 58 472–486. 10.1037/0022-3514.58.3.472 2324938

[B50] Papadatou D. Giannopoulou I. Bitsakou P. Bellali T. Talias M. A. Tselepi K. (2012). Adolescents’ reactions after a wildfire disaster in Greece. *J. Trauma Stress* 25 57–63. 10.1002/jts.21656 22298431

[B51] Pihet S. Moses Passini C. Eicher M. (2017). Good and bad days: fluctuations in the burden of informal dementia caregivers, an experience sampling study. *Nurs. Res.* 66 421–431. 10.1097/NNR.0000000000000243 29095373

[B52] Pritchard C. Gow K. M. (2012). “Coping self-efficacy and psychological distress in flood victims,” in *Individual trauma: Recovering from Deep Wounds and Exploring the Potential for Renewal*, eds Gow K. M. Celinski M. J. (New York, NY: Nova Science), 207–219.

[B53] Psarros C. Theleritis C. Economou M. Tzavara C. Kioulos K. T. Mantonakis L. (2017). Insomnia and PTSD one month after wildfires: evidence for an independent role of the “fear of imminent death.”. *Int. J. Psychiatry Clin. Pract.* 21 137–141. 10.1080/13651501.2016.1276192 28084115

[B54] Rothbaum B. O. Hodges L. F. Ready D. Graap K. Alarcon R. D. (2001). Virtual reality exposure therapy for Vietnam veterans with posttraumatic stress disorder. *J. Clin. Psychiatry* 62 617–622. 10.4088/JCP.v62n0808 11561934

[B55] Rothbaum B. O. Schwartz A. C. (2002). Exposure therapy for posttraumatic stress disorder. *Am. J. Psychother.* 56 59–75. 10.1176/appi.psychotherapy.2002.56.1.59 11977784

[B56] Smith B. W. Freedy J. R. (2000). Psychosocial resource loss as a mediator of the effects of flood exposure on psychological distress and physical symptoms. *J. Trauma Stress* 13 349–357. 10.1023/A:1007745920466 10838680

[B57] Stacklies W. Redestig H. Scholz M. Walther D. Selbig J. (2007). pcaMethods—A bioconductor package providing PCA methods for incomplete data. *Bioinformatics* 23 1164–1167. 10.1093/bioinformatics/btm069 17344241

[B58] St. Louis-Sanchez M. (2012). *Waldo Canyon Fire: Tally of Destruction Remains Imprecise.* Colorado Springs, CO: Colorado Springs Gazette.

[B59] Thoresen S. Tambs K. Hussain A. Heir T. Johansen V. A. Bisson J. I. (2010). Brief measure of posttraumatic stress reactions: impact of Event Scale-6. *Soc. Psychiatry Psychiatr. Epidemiol.* 45 405–412. 10.1007/s00127-009-0073-x 19479171

[B60] Wadsworth M. E. Santiago C. D. Einhorn L. (2009). Coping with displacement from Hurricane Katrina: predictors of one-year post-traumatic stress and depression symptom trajectories. *Anxiety Stress Coping* 22 413–432. 10.1080/10615800902855781 19343597

[B61] Weathers F. W. Blake D. D. Schnurr P. P. Kaloupek D. G. Marx B. P. Keane T. M. (2013). *The Life Events Checklist for DSM-5 (LEC-5).* Available online at: https://www.ptsd.va.gov/professional/assessment/te-measures/life_events_checklist.asp (accessed March 7, 2018).

[B62] Weiss D. S. (2004). “The impact of event scale-revised,” in *Assessing Psychological Trauma and PTSD*, 2nd Edn, eds Wilson J. P. Keane T. M. (New York, NY: Guilford Press), 168–189.

[B63] Witkiewitz K. van der Maas H. L. J. Hufford M. R. Marlatt G. A. (2007). Nonnormality and divergence in posttreatment alcohol use: reexamining the Project MATCH data. *J. Abnorm. Psychol.* 116 378–394. 10.1037/0021-843X.116.2.378 17516769 PMC2048690

[B64] Xu Y. Chen X. Yu B. Joseph V. Stanton B. (2017). The effects of self-efficacy in bifurcating the relationship of perceived benefit and cost with condom use among adolescents: a cusp catastrophe modeling analysis. *J. Adolesc.* 61 31–39. 10.1016/j.adolescence.2017.09.004 28946075

[B65] Yu B. Chen X. Stanton B. Chen D.-G. Xu Y. Wang Y. (2018). Quantum changes in self-efficacy and condom-use intention among youth: a chained cusp catastrophe model. *J. Adolesc.* 68 187–197. 10.1016/j.adolescence.2018.07.020 30118949 PMC6157611

